# Functionally active cross-linked protein oligomers formed by homocysteine thiolactone

**DOI:** 10.1038/s41598-023-32694-2

**Published:** 2023-04-06

**Authors:** Kritika Kumari, Gurumayum Suraj Sharma, Akshita Gupta, Khuraijam Surjalal Singh, Laishram Rajendrakumar Singh

**Affiliations:** 1 Dr. B. R. Ambedkar Center for Biomedical Research, University of Delhi, Delhi, 110007 India; 2 Department of Botany, Bhaskaracharya College of Applied Sciences, University of Delhi, New Delhi, 110075 India

**Keywords:** Biochemistry, Biophysics

## Abstract

Deposition of high-order protein oligomers is a common hallmark of a large number of human diseases and therefore, has been of immense medical interest. From the past several decades, efforts are being made to characterize protein oligomers and explore how they are linked with the disease pathologies. In general, oligomers are non-functional, rather cytotoxic in nature while the functional (non-cytotoxic) oligomers are quite rare. In the present study, we identified new protein oligomers of Ribonuclease-A and Lysozyme that contain functionally active fractions. These functional oligomers are disulfide cross-linked, native-like, and obtained as a result of the covalent modification of the proteins by the toxic metabolite, homocysteine thiolactone accumulated under hyperhomocysteinemia (a condition responsible for cardiovascular complications including atherosclerosis). These results have been obtained from the extensive analysis of the nature of oligomers, functional status, and structural integrity of the proteins using orthogonal techniques. The study implicates the existence of such oligomers as protein sinks that may sequester toxic homocysteines in humans.

## Introduction

Protein misfolding and aggregation are of immense medical interest since deposition of toxic protein aggregates has a close association with several human diseases^1^. In general, misfolded protein species either undergo enhanced degradation or end up with high-order oligomers. Such toxic high-order oligomers are then deposited in the cellular compartments or extracellular matrices and become the bottleneck for the development of disease pathologies^2,3^. Several protein aggregates have been identified in vitro and in vivo which include disordered aggregates, small oligomers, amyloid fibrils, native-like aggregates, etc. Among the disordered aggregates, amorphous deposits and folding aggregates are in vitro aggregates while inclusion bodies are the classic examples of in vivo species^4^. Amyloid fibrils which are indeed, highly ordered aggregates are formed by cross β-sheets^5^ and are considered to be the emblematic cause of neurodegenerative diseases^6^. Amorphous protein aggregates have also been associated with cataract (caused due to aggregation of α-crystallin), renal failure (caused due to aggregation of immunoglobulin), Parkinson's disease (caused due to α-synuclein aggregation), etc.^7–10^. Small oligomers are generated from the transient aggregation-prone intermediates (APIs) which are the precursors of amyloid fibrils or disordered aggregates^11,12^.

In hyperhomocysteinemia (a condition characterised by the elevated level of plasma homocysteine), formation of different protein oligomers result in the toxic gain of function and consequent increase in oxidative stress. These oligomers are produced due to the covalent modification of proteins by homocysteine thiolactone (HTL). HTL is a highly reactive cyclic thioester of Hcy formed by methionyl-tRNA synthetase in an error-editing reaction. HTL has the potential to form covalent bond with *ε*-amino group of lysine residues in proteins (a process called protein *N*-homocysteinylation). This modification generates a free sulfhydryl (SH–) group in the polypeptide which can form disulfide bond with another SH– group on other polypeptides resulting in the formation of cross-linked oligomers^13^. The formation of such cross-linked oligomers in hyperhomocysteinemia is considered to be a basic cause of oxidative stress^14^, proteotoxicity^15^, and/or cytotoxicity^16^. Although advances have been made in understanding oligomers formed by various modifications, HTL-induced cross-linked oligomers have not been studied in detail. Therefore, the nature, structure, and functional behavior of the HTL-induced cross-linked oligomers remain completely unelucidated. In the present study, we attempted to systematically analyze HTL-induced cross-linked protein oligomers formed by Ribonuclease-A (RNase-A) and Lysozyme (Lyz). We have chosen these proteins because, despite covalent modification by HTL, these proteins do not render functional loss^15^. We discovered two novel HTL-induced cross-linked oligomers that contain functionally active fractions and therefore, implicate their role in hyperhomocysteinemia.

## Results

### HTL induces covalent modification of RNase-A and Lyz

In order to check if RNase-A and Lyz undergo covalent modification by HTL, we have treated both the proteins with HTL and analysed the modification by measuring free –SH content. We have chosen 4 different concentrations (250, 500, 750, and 1000 µM) of HTL as these concentrations are conventionally used in previous studies with respect to protein N-homocysteinylation^16–18^. It is seen in Table 1 that as compared to the unmodified controls, there is a large increase in the total -SH content of the modified proteins in a HTL concentration-dependent manner indicating that the proteins are covalently modified. It is also seen in the figure that samples incubated for 48 h have more total -SH content (at all HTL concentrations) relative to samples incubated for 24 h.

**Table 1:** Free sufhydryl content of HTL-modified proteins: Total free -SH content of HTL- modified RNase-A and Lyz taken at intervals of 24 h and 48 h.

	Total -SH content, mol/mol × 1000	
HTL concentration (µM)	24 h	48 h
**RNase-A**		
0	88.09 ± 14.17	101.72 ± 8.77
250	249.76 ± 33.75	344.26 ± 27.13
500	486.02 ± 24.30	769.54 ± 21.60
750	681.78 ± 34.08	1113.8 ± 23.62
1000	911.30 ± 45.56	3375.18 ± 19.57
**Lyz**		
0	136.80 ± 12.95	145.88 ± 03.60
250	158.29 ± 35.85	324.02 ± 50.25
500	288.01 ± 28.65	691.24 ± 53.64
750	446.42 ± 27.07	936.05 ± 43.05
1000	612.03 ± 21.60	1216.87 ± 30.24

### Covalently-modified RNase-A and Lyz form large-size spherical oligomers

**Figure 1:**
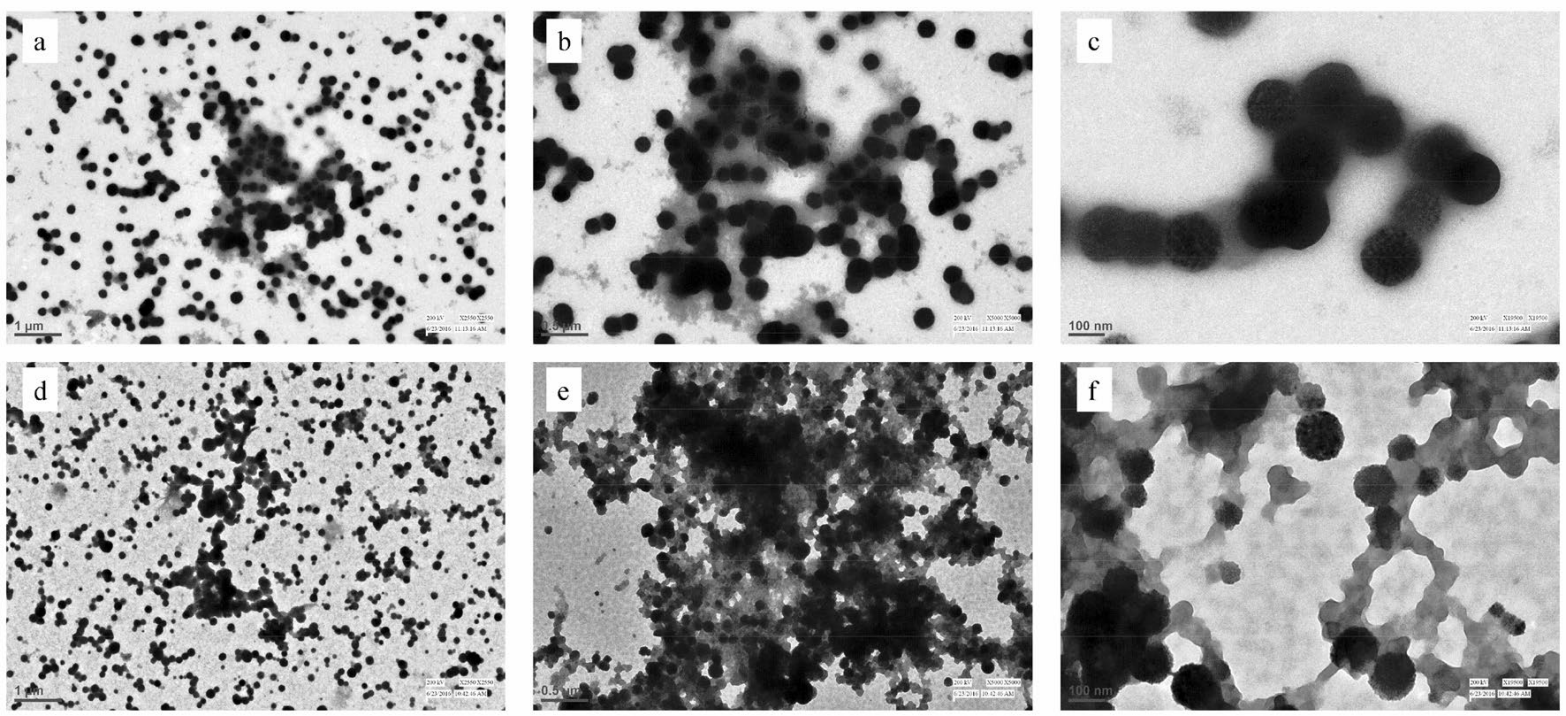
Morphology of HTL-modified proteins: Transmission electron micrographs images of modified RNase-A (Panel **a**, **b** and **c**) and Lyz (Panel **d**, **e**, and **f**). Panels (**a**) and (**d**) are micrographs at magnifications 1 µm; Panels **b** and **e** are at 0.5 µm; and Panels (**c**) and (**f**) are at magnifications of 100 nm respectively. µm represents scalar unit micrometer. The concentration of HTL used was 1000 µM.

To investigate the nature of oligomers formed by the HTL-modified proteins, first of all, we carried out HTL-induced modification of the proteins for 7 days and analysed the nature of oligomers using dynamic light scattering (DLS) (Table 2). Representative raw data (size distribution by volume) of day 1 and day 7 are also shown in Figure S1. It is seen in Table 2 that for at least 2 days, both proteins remain in the monomeric state as indicated by no significant alteration in the hydrodynamic diameter of the proteins. It is important to note that in case of RNase-A, there is presence of both monomer and oligomer on day 2 at the highest HTL concentration. Subsequently, upon extending the incubation period, there is an increase in the size of the oligomers in a day-dependent manner (at each concentration) (Table 2). It is also seen in the table that although many of the modified samples comprise single oligomeric species, some of the samples contain heterogenous species in both proteins. On day 7, there is the existence of oligomers ranging from around 500–1500 nm in size, beyond which there is visible precipitation. TEM electro-micrograph further revealed that the morphology of the oligomer is spherical (Fig. 1) for both proteins.

**Table 2:** Hydrodynamic diameter of HTL-modified protein samples: Hydrodynamic diameter (in nm) of RNase-A and Lyz modified with 250 µM, 500 µM, 750 µM, and 1000 µM HTL at 24 h intervals up to 7 days measured using DLS.

HTL					
Days	0 µM	250 µM	500 µM	750 µM	1000 µM
**RNase-A**					
1	3.2 ± .1	3.5 ± .3	4.1 ± .4	3.6 ± .3	3.6 ± .4
2	3.7 ± .3	4.1 ± .4	4.2 ± .6	3.6 ± .8	175.7 ± 9.1, 3.7 ± .1
3	3.6 ± .3	136.6 ± 9, 395.9 ± 36	235 ± 13	205.6 ± 11.5	316.0 ± 27.1
4	3.1 ± .2	156.6 ± 7.8	502.2 ± 45	507.6 ± 39.5	494.5 ± 64.0
5	3.2 ± .3	97.8 ± 4, 233.9 ± 18	548.0 ± 35.6	485.5 ± 31.2	551.6 ± 42.3
6	3.5 ± .3	546.0 ± 54	626.8 ± 35.9	571 ± 38.7	518.3 ± 43.6
7	3.7 ± .3	708.5 ± 49	679.8 ± 59.3	694.7 ± 48.3, 1621 ± 182	503.2 ± 67.8, 1515 ± 167
**Lyz**					
1	3.4 ± .3	3.9 ± .3	3.7 ± .3	4.2 ± .3	3.8 ± .3
2	3.4 ± .3	3.6 ± .2	3.8 ± .3	3.5 ± .2	3.8 ± .4
3	3.6 ± .2	3.9 ± .3	3.7 ± .4	137.6 ± 7.4, 86.6 ± 5.5	192.5 ± 12.5, 231.2 ± 17.6
4	3.3 ± .5	107.0 ± 5.3	252.7 ± 18.8	304.6 ± 21.4	275.1 ± 21.7, 105.2 ± 10
5	3.9 ± .1	97.7 ± 4.6	223.6 ± 16.41, 521.8 ± 63.5	426.5 ± 28.1	454.9 ± 45.6
6	3.7 ± .3	546.0 ± 54.5, 1314 ± 167	578.9 ± 78.8	603.7 ± 31.8	507.3 ± 36.0
7	3.5 ± .2	558.9 ± 57.4, 1308 ± 146	727.1 ± 120.6	432.5 ± 31.6, 1262 ± 89	705.4 ± 79.4

### HTL-modified RNase-A and Lyz exhibit exceptionally longer lag phase

**Figure 2:**
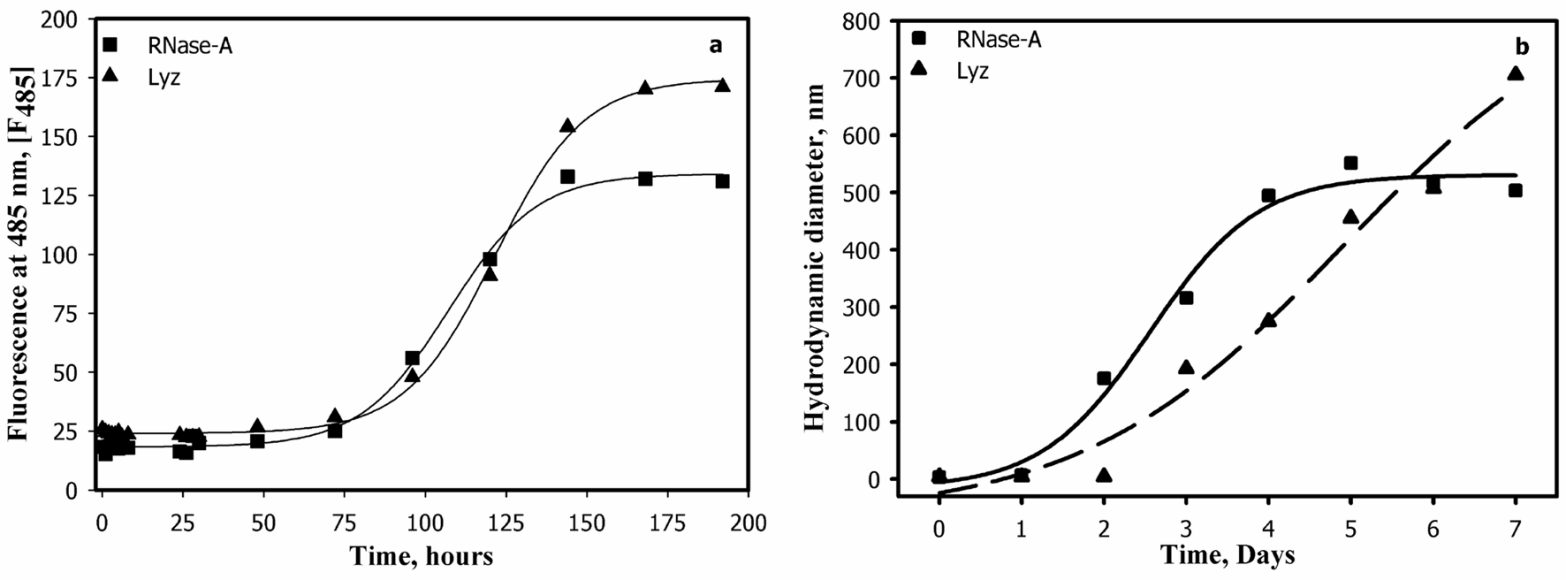
Aggregation kinetics profiles of HTL-modified proteins: Time-dependent aggregation curves of HTL-modified RNase-A and Lyz (Panel **a**) taken at intervals of 24 h monitored using ThT dye. HTL concentration was kept constant at 1000 µM for all the experiments. Panel (**b**) represents the aggregation kinetic curve of HTL-modified RNase-A and Lyz obtained by plotting hydrodynamic radii versus time using values obtained from Table 1.

In general, protein aggregation comprises of a lag phase (the nucleation step characterised by the subtle conformational alteration in the native state resulting in the formation of API), log phase (represented by the formation of oligomers of different sizes) and stationary phase (characterised by the formation of final mature oligomers). In order to investigate the aggregation behaviors of HTL modified Lyz and RNase-A, we have measured time-dependent aggregation kinetics of both the proteins by monitoring ThT fluorescence at different time intervals and the relative fluorescence was plotted against time as shown in Fig. 2. It is seen in Fig. 2 that both proteins exhibit conventional aggregation kinetics as exemplified by the presence of sigmoidal curves comprising of a lag phase, log phase, and mature oligomers. It is also seen in the figure that RNase-A and Lyz initiate oligomer formation by 77 and 83 h and mature oligomers are obtained at 168 and 145 h respectively. Each aggregation kinetic curve has been analysed for kinetic parameters; magnitude of final oligomer (*I*_*f*_), apparent rate constant (*k*_app_) and lag time (*t*_lag_) using appropriate equations and are given in Table S1. It is seen in this table that both proteins exhibit variable *t*_lag_. It may also be noted that Lyz and RNase-A comprise of exceptionally long lag phase (*t*_lag_: 76–85 h), the rate of oligomerization step (*k*_app_) is extremely slow. There are also differences in the magnitude of the final oligomers (*I*_*f*_) for each protein. Figure 2b also shows the aggregation kinetic curves of RNase-A and Lyz obtained by plotting hydrodynamic diameters obtained on each day (from days 1–7). It is seen in this figure that the proteins also exhibit similar sigmoidal aggregation curves and it took 5–7 days to form mature aggregates.

### Oligomers formed by modified RNase-A and Lyz contain functionally active fractions

To investigate the functional status of the oligomer-containing samples, we have made systematic measurements of enzyme activity of the modified RNase-A and Lyz from day 1–7. Table 3 represents the change in percent activity of RNase-A and Lyz upon modification with 250 µM, 500 µM, 750 µM, and 1000 µM concentrations of HTL. Surprisingly, there was no significant loss of activity of the modified proteins for up to 2 days at all the HTL concentrations. It may also be noted that in all samples from days 3–7, there was an observed decrease in enzyme activity in a day-dependent manner. It may be noted that there was no complete loss of activities even up to day 7 wherein mature oligomers are formed (Fig. 2). There was retainment of activity around 59% and 39% in the case of RNase-A and Lyz respectively at the highest concentration of HTL. The presence of activity in the samples that even contain mature oligomers indicates that oligomers consist of functionally active fractions.

**Table 3:** Enzyme activity of HTL-modified RNase-A and Lyz: percent enzyme activity of HTL-modified RNase-A and Lyz at intervals of 24 h for seven days. Errors in enzyme activities of RNase-A and Lyz are 5–8% and 4–9% respectively.

HTL					
Days	0 µM	250 µM	500 µM	750 µM	1000 µM
**RNase-A**					
0	100	100	100	100	100
1	100	98.78 ± 2.9	97.37 ± 2.2	98.45 ± 3	97.49 ± 2.3
2	96	100.79 ± 1.7	97.67 ± 2.9	94.49 ± 2.1	95.30 ± 1.9
3	98	96.49 ± 1.2	95.17 ± 2.3	95.89 ± 1.6	88.84 ± 1.2
4	93	100.74 ± 1.01	93.39 ± 1.9	90.12 ± 1.5	78.09 ± 1
5	94	98.85 ± 1.1	91.78 ± 1.2	89.39 ± 1.6	75.60 ± 2
6	92	81.72 ± 2.1	78.92 ± 1.3	72.04 ± 3.1	64.50 ± 2.6
7	93	77.98 ± 2.3	61.62 ± 1.9	57.01 ± 1.8	58.98 ± 1
**Lyz**					
0	99 ± 0.8	100	100	100	100
1	100 ± 1.4	96.82 ± 1.2	98.23 ± 1.9	97.79 ± 1.5	98.91 ± 1.6
2	99 ± 1.4	94.18 ± 1.3	95.83 ± 2	94.14 ± 1.5	94.21 ± 1.7
3	97 ± 2.4	92.13 ± 2	89.65 ± 1.7	85.67 ± 3	78.98 ± 2
4	99 ± 1.2	78.37 ± 3	77.89 ± 3.1	73.37 ± 2.8	70.59 ± 3.1
5	98 ± 1.7	65.88 ± 3.1	61.93 ± 3	56.90 ± 2	51.49 ± 2.2
6	96 ± 2.6	58.66 ± 2	55.45 ± 1.7	53.71 ± 1.8	42.59 ± 1.9
7	95 ± 1.9	39.19 ± 2.4	39.98 ± 3.5	35.19 ± 2	38.63 ± 2

### HTL modification does not influence the structural integrity of the proteins

**Figure 3:**
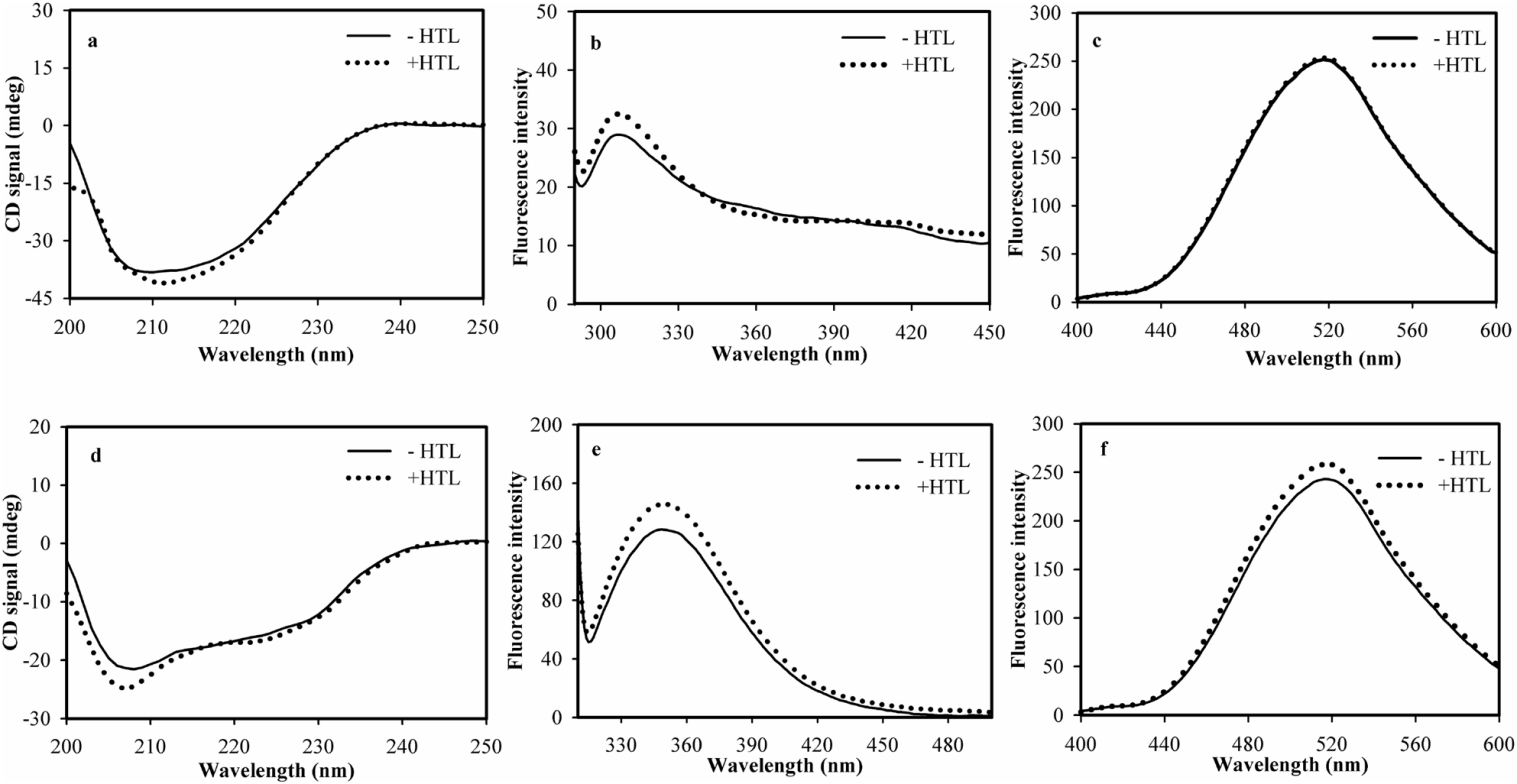
Conformational changes of the HTL-modified RNase-A and Lyz. Far UV CD spectra (left panel), intrinsic fluorescence spectra (middle panel), and ANS-binding spectra (right panel) of HTL-modified RNase-A and Lyz. The structures represent modified samples of day 7.

Conformational analysis of the HTL-modified RNase-A and Lyz was carried out using different spectroscopic tools (Fig. 3). Figure 3a,d shows the far-UV CD spectra of the HTL-modified proteins at day 7. It is seen in this figure that there is an apparent change in the secondary structural elements of the modified proteins. Systematic evaluation of the individual secondary structural components revealed that there is no change in the α-helix, β-sheet, but a slight alteration in the random coil component (Fig. S2). Tryptophan fluorescence spectra of the modified proteins suggest that there is an subtle increase in the environment of the tryptophan as indicated by the observed hyperchromicity at 320 and 350 nm in case of RNase-A and Lyz respectively (Fig. 3b,e). Such a partial change in the micro-environment of tryptophan might have affected the overall packing of the native state leading to the exposition of hydrophobic groups to the solvent. 8-anilino-1-naphthalene sulfonic acid (ANS) is a dye that specifically binds to the exposed hydrophobic groups and therefore, could be used to detect the exposed hydrophobic groups. No blue-shift and lack of hyperchromicity indicate the absence of ANS binding to the modified proteins. Taken together, the results rule out the possibility of large alteration in the tertiary structure due to HTL-induced modification in both proteins.

### HTL-induced oligomer is reversible upon Dithiothreitol (DTT) treatment

**Figure 4:**
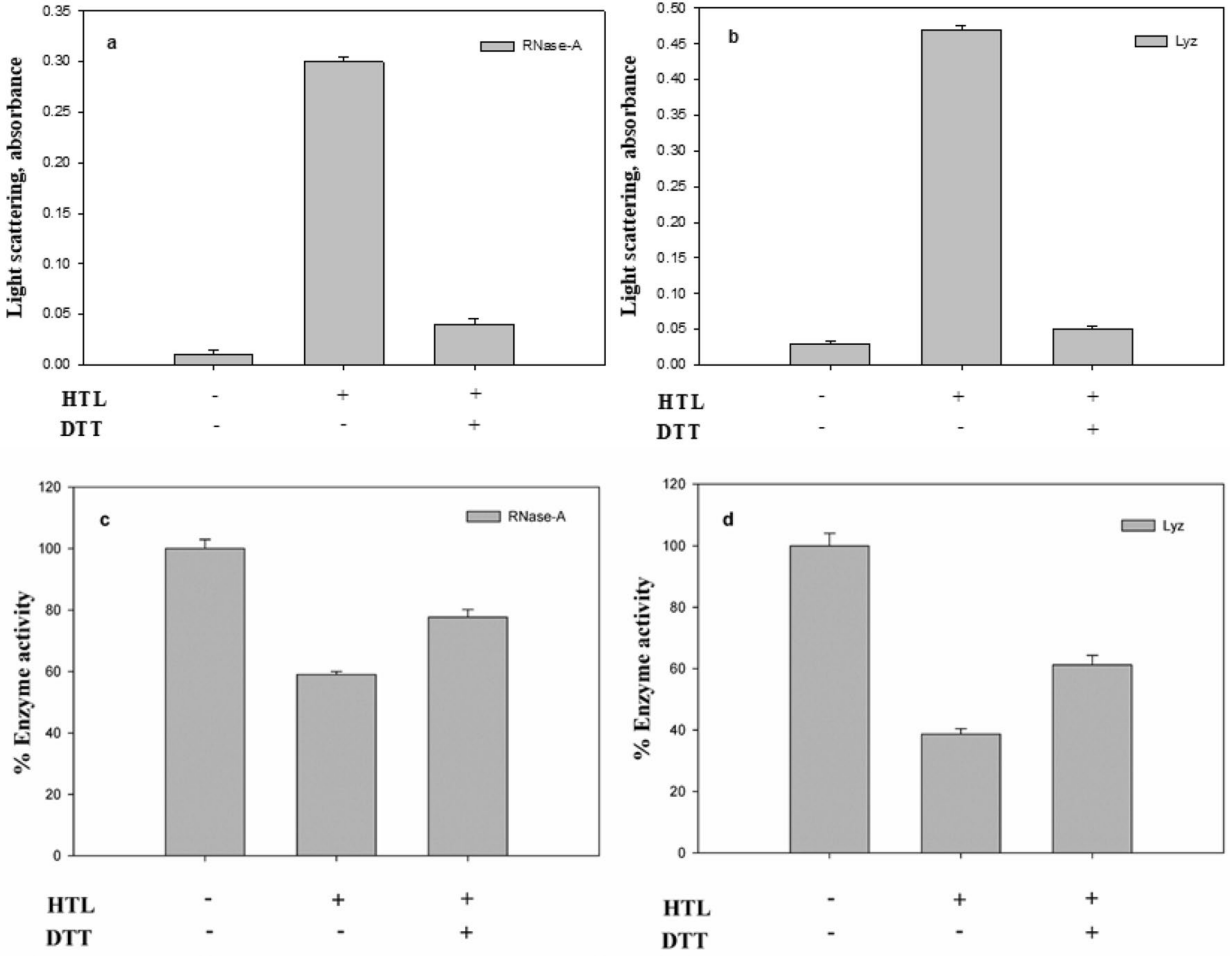
Behavior of HTL-modified proteins in presence of DTT: Light scattering intensity of oligomers formed by HTL-modified RNase-A (panel **a**) and Lyz (panel **b**) samples of day 6 upon addition of DTT. Enzyme activity of oligomers formed by HTL-modified RNase-A (panel **c**) and Lyz (panel **d**) samples of day 6 upon addition of DTT.

It was also important to check the nature of the oligomer developed due to the HTL-induced modification of the proteins. Theoretically, there is generation of a free -SH upon covalent modification of a protein that may eventually form disulfide bonds with another polypeptide creating a cross-linked oligomer. We have, therefore, treated the oligomers formed on day 6 with DTT and examined the oligomeric status by measuring light scattering intensity (Fig. 4). It is seen in this figure that there is an increase in light scattering intensity of the HTL-modified protein samples indicating the presence of large protein oligomers. Upon treatment with DTT, it decreases nearly identically to that of the unaggregated modified samples of day 2 (Fig. 4a). Activity measurements also indicate that there is loss of enzyme activity by 40% and 60% respectively in case of HTL modified RNase-A and Lyz. Upon addition of DTT, there is an increase in the enzyme activity of the modified RNase-A and Lyz by around 20% and 27% respectively relative to the modified controls (Fig. 4b). The results indicate that the oligomers formed by both proteins are disulfide-linked and reversible.

### HTL-grown protein oligomers are not cytotoxic

**Figure 5:**
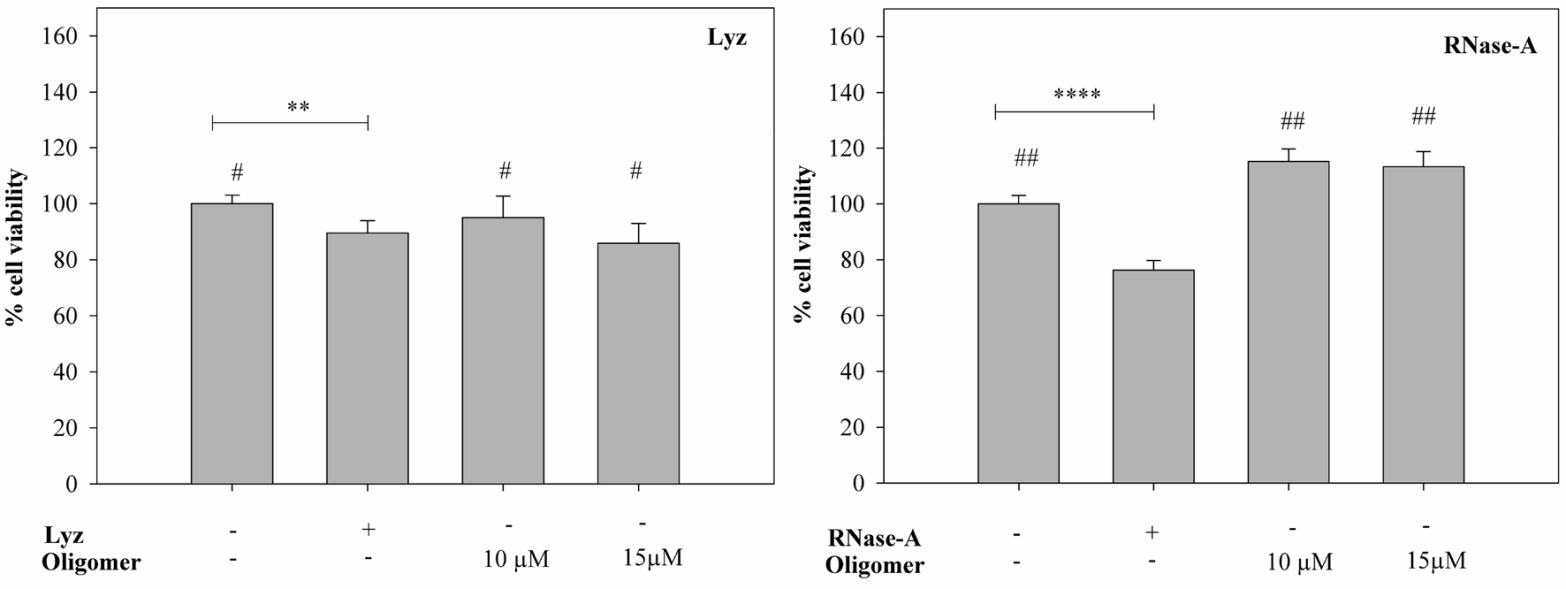
Cytotoxic behavior of HTL-induced protein oligomers on HeLa cells: Percent viability of cells in the presence and absence of Lyz oligomers formed on day 6 (left panel) and RNase-A oligomers (right panel).

To investigate the cytotoxic potential of the protein oligomers induced by the covalent modification of Lyz and RNase-A by HTL, we treated the HeLa cells with two different concentration of oligomers and measured percent cell viability using MTT assay. It is seen in Fig. 5 that unmodified Lyz exhibits little decrease in cell viability (around 10%). However, there is no significant change in cell viability between the native and HTL-modified Lyz. Additionally, in the presence of 10 µM HTL concentration, there was an apparent increase in cell viability that is identical to that of the untreated cells. On the other hand, unmodified RNase-A is cytotoxic because there was significant reduction (around 30%) in the cell viability. However, its oligomers exhibit proliferative effect as there was an increase in the percent viable cells (13–15%). The results indicate that the HTL-grown oligomers of RNase-A and Lyz are not cytotoxic to HeLa cells.

## Discussion

**Figure 6:**
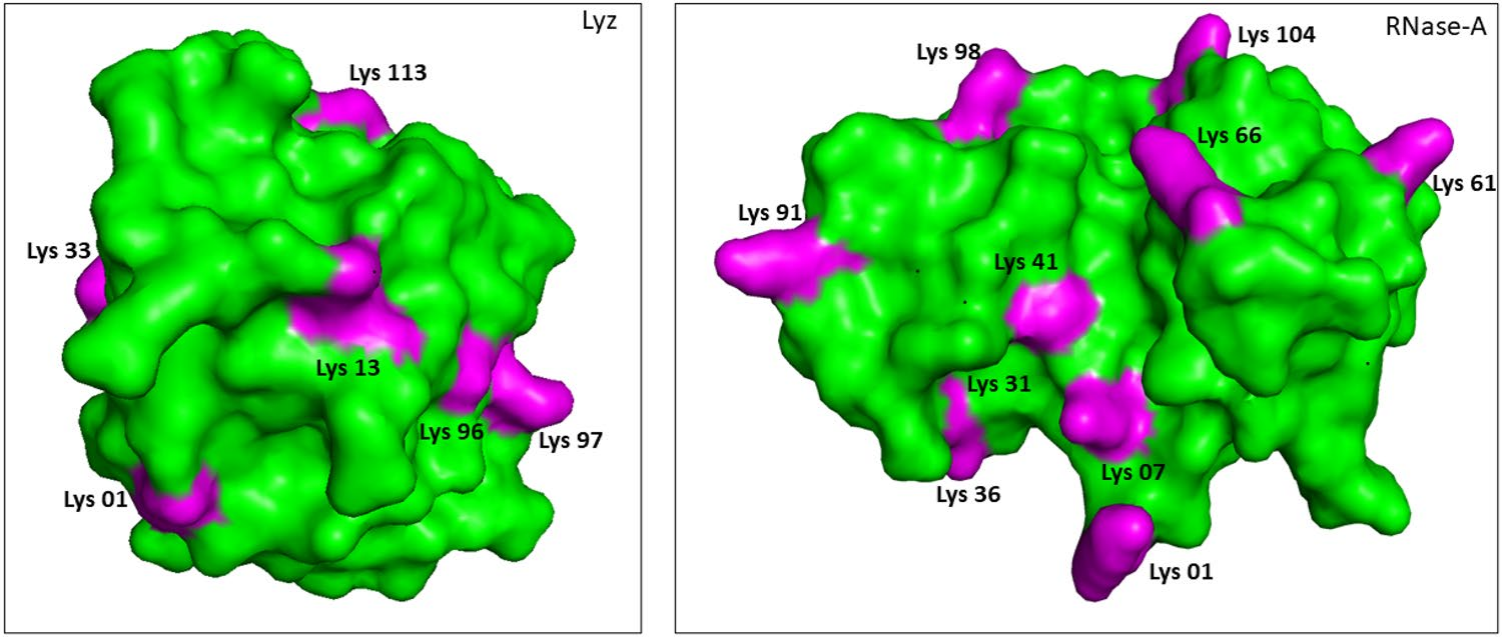
3D structures of Lyz (left panel) and RNase-A (right panel) indicating the positions of the lysine residues.

HTL commonly induces covalent modification of lysine residues in a polypeptide chain that results in the generation of free SH- group(s), which may eventually form disulfide bonds with SH-group(s) on other polypeptides leading to the formation of cross-linked oligomers^19^. To analyze the oligomers formed by HTL modification in RNase-A and Lyz, first of all, we carried out systematic measurement of the hydrodynamic radius of the modified proteins using DLS (Table 2). Three important observations from our DLS studies are (i) for the first 2 days, modified samples of both the proteins are completely devoid of oligomers, (ii) oligomers appeared up in the samples incubated for days 3–7, and (iii) large size oligomers ranging from around 500–1500 nm (i.e., 145–450 fold bigger than the unmodified control) are present in samples of day 7. It is also evident in Table 1 that the protein samples undergo extensive modification by HTL at 24 and 48 h. Therefore, absence of oligomers in the samples of day 1 and 2 is not because of the lack of modifications by HTL. Further observations revealed that the oligomerization process appears to follow a “primary nucleation dependent” mechanism^20^ because oligomer(s) of sufficient size (critical nucleus) is obtained on day 3 followed by the growth of the oligomer in a day-dependent manner (Fig. 2). Commonly, spherical or amorphous aggregates are formed if the starting conformer is helical in structure while amyloid fibrils are generated if it is β-sheet^21^. To explore this possibility, we have intentionally analyzed the morphology of the oligomers formed on day 7. It is evident in Fig. 1 that despite of having different native state structures (RNase-A, α/β; Lyz, α-type), HTL modification induces similar spherical oligomers in both the proteins. Examination of the protein sequences further revealed that RNase-A and Lyz contain 6 and 10 lysine residues respectively. Interestingly, all these residues are exposed to the surface and are evenly distributed throughout the surface in both the proteins (Fig. 6) indicating that there are common features between the two proteins in terms of lysine distribution and is the reason why common spherical oligomers are generated. It may also be noted that results on TEM images (Fig. 1) appear to contradict the previous results obtained on the nature of protein oligomers using ThT dye (Fig. 2) because if oligomers are spherical in nature, they should not react with amyloid specific ThT dye. Additionally, aggregation of HTL-modified RNase-A and Lyz is much unlike the formation of amyloid fibrils with cross-beta structure, whereby most protein molecules need to partially unfold before they adopt a β-sheet structure. ThT molecules are also known to get embedded into the ridges of amyloid fibrils, which allows them to exhibit fluorescence. However, ThT is also known to bind to the hydrophobic pockets of non-amyloidogenic proteins^22–24^. It has been reported to bind even on the alpha-helical proteins including acetylcholinesterase^25^ and albumins^26^. Although both RNase-A and Lyz do not undergo conformational change due to the modification (Fig. 3), formation of oligomers might have created hydrophobic contacts favorable for ThT binding. In support, such binding behavior of ThT has been reported in the case of the oligomerization of albumin^26^.

It is commonly believed that initiation of oligomer formation involves change in the native state structural integrity^27^. However, this is not the case in the present study because there are no significant differences in the secondary and tertiary structures of the modified and unmodified proteins (Fig. 3). Taken together, the results indicate that the oligomers formed by HTL modification are native-like cross-linked oligomers. In addition to Lyz and RNase-A, formation of native-like oligomers due to HTL modification has also been previously reported in case of serum albumin^28^.

As mentioned above, HTL modification generates a disulfide-linked oligomer. Therefore, it is also important to verify that the oligomers formed are cross-linked via disulfide linkages. If the oligomers are disulfide-linked, upon treatment with DTT, the oligomers should be reversible to their monomeric species. For this, we have intentionally treated the modified samples of day 6 with DTT and analysed them for protein oligomers (by measuring light scattering intensity) and functional status (Fig. 4). Disappearance of oligomers upon DTT treatment (Fig. 4a) and reversal of enzyme activity by around 15–21% (Fig. 4b) further confirms that oligomers are disulfide-linked.

In general, protein aggregation process comprises of 3 different steps: nucleation, oligomerization, and stationary phase. In the nucleation step, native proteins undergo certain conformational changes and consequently convert to an API. The so-formed API drives the generation of high-order oligomers that ultimately ends up with mature aggregates^29^. To investigate the aggregation behavior of the modified proteins, we measured the aggregation kinetics of Lyz and RNase-A (Fig. 2). It was observed that RNase-A and Lyz exhibit a lag phase (*t*_lag_) of around 76–83 h, and mature oligomers are formed at day 7 (Table S1) indicating a slow oligomerization step. Furthermore, there is large delay in the oligomerization step as the *K*_app_ was around 0.073 h for both the proteins. Thus, overall data indicate that longer *t*_lag_ and very slow *K*_app_ might be responsible for an exceptionally longer period (7 days) to form mature oligomers.

One generally held belief is that HTL-induced protein modification and the eventual formation of protein oligomers are responsible for the functional deficiency of enzymes^13,30^. Therefore, to investigate for the functional alterations of the HTL-modified protein samples, we have analysed the activity status of the modified proteins up to 7 days. The observation that samples of day 1 and 2 are completely resistant to functional change (Table 3) and yet devoid of any oligomers indicates (Table 1) that modification does not affect the functionality of the enzymes. No effect on enzyme activity despite modification, might be due to the absence of lysine residues in the active sites and unaltered native state (Fig. 3). To date, there has been no literature that HTL-modified proteins renders no functional loss. Therefore, observations on the functional status of the RNase-A and Lyz are unique among the various HTL-modified proteins.

**Figure 7:**
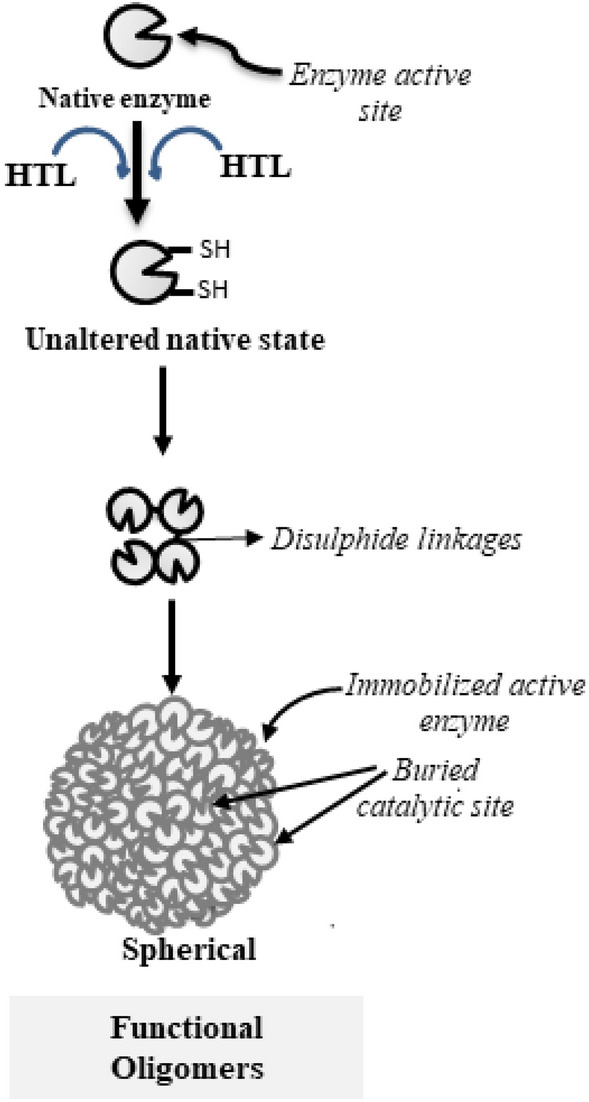
Image depicting the presence of functional active fractions in the oligomer. The diagram has been shown as 2D.

**Figure 8:**
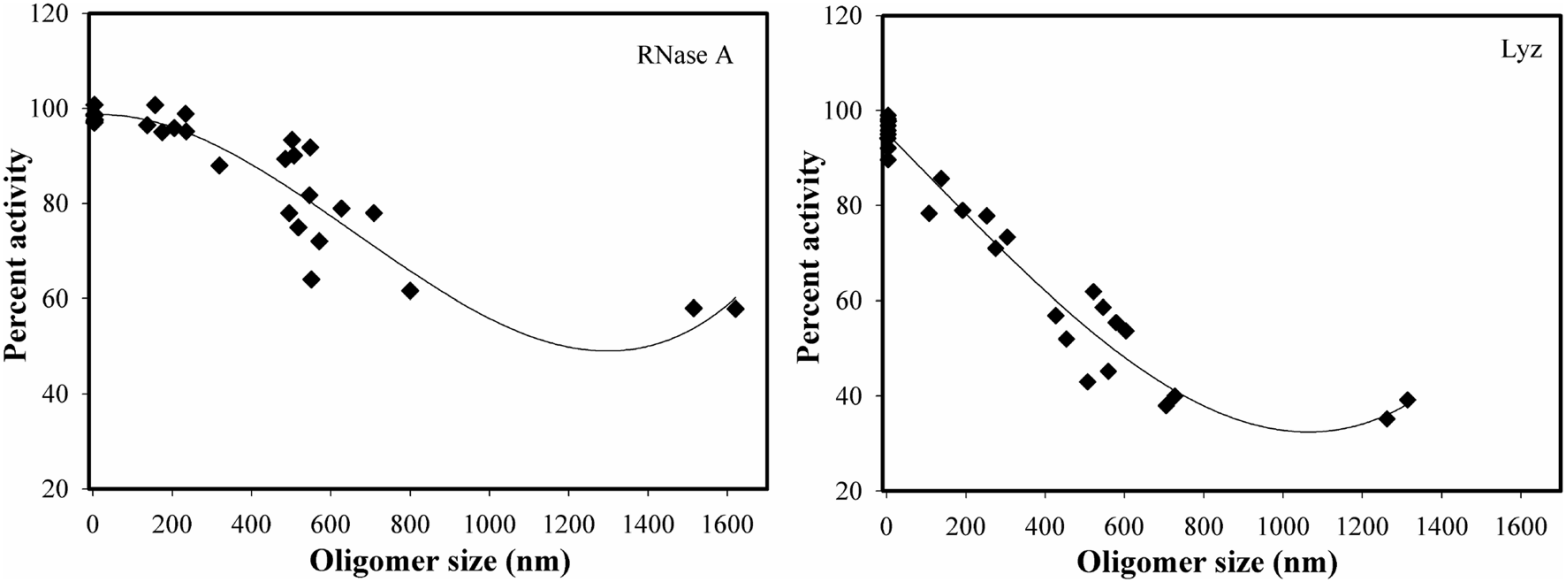
Relation between functional activity and size of oligomers: Plot of enzyme activity versus oligomer size of HTL-modified RNase-A (left panel) and Lyz (right panel).

A systematic analysis of the functional activity status of the modified protein samples (Table 3) and oligomer sizes (Table 2) of day 3–7 further revealed a apparent correlation between the growth of the protein oligomers and the reduction in enzyme activities. Since, we use excess HTL concentration relative to the proteins (around 1:1000, protein to HTL molar ratio), there should not be any unmodified protein molecules in the samples and conversely, all modified protein molecules should cross-link to form oligomers. Therefore, the oligomers generated in all samples are expected to be non-functional. However, this is not true in our case. Although, the oligomer size increases in a day-dependent manner (Table 2), we observed retainment of activity fractions in all the protein samples from days 3–5 and even in samples of days 6–7 (Table 3) wherein mature oligomers are already formed. The results indicate the possibility that the oligomers are functionally active. Perhaps, as a part of the assembly of the modified proteins into larger species, many of the monomeric or smaller oligomers are buried into the core of oligomeric assembly, and hence their active sites remain inaccessible to the substrate. Furthermore, some of the modified protein fractions are on the surface whose active sites are exposed to the solvent and immobilized on the oligomer surface as shown in Fig. 7. If this is the case, upon reaching a sufficient size, the activity should either be zero or there should be an activity trade-off (level-off) mechanism as the oligomer can no longer grow in size. For this, we have intentionally plotted oligomer size versus enzyme activity (Fig. 8**)**. It is evident in this figure that there is an apparent relationship between the oligomer size and loss in functional activity. However, when the size of the oligomer is sufficiently bigger, the activity of the oligomeric species levels off (but not zero) and does not decrease thereafter, confirming that there is an oligomer-activity trade-off. However, it is worthwhile to mention that presence of traces of unmodified proteins might also be responsible for the retainment of activity. To rule out for this possibility, we have further analysed the total –SH content of the modified protein samples on each day (Table S2). It is evident in this table that there is a gradual increase in the total -SH content upto day 6 but beyond which it levels off indicating that there is no further modification after day 6. The results led us to believe that there was complete absence of unmodified protein molecules at day 7. Taken together, we conclude that oligomers contain functionally active fractions.

We were further interested to investigate if these functional HTL-grown oligomers are cytotoxic to the cells. It is evident in Fig. 5 that there are two different consequences on the growth of the HeLa cells in the presence of the oligomers formed via N-homocystinylation. We observed no significant difference between the percent viability of HeLa cells in the presence of unmodified and HTL-modified Lyz. However, there is an apparent increase in the proliferation of the cells in the case of modified RNase-A as evident by the 13–15% increase in the number of viable cells. Altogether, the treatments have not largely altered the cell viability indicating that the HTL-grown oligomers of Lyz and RNase-A are apparently non-cytotoxic.

At present, we do not have any data to support the physiological significance of the presence of such functional cross-linked oligomers formed by HTL modification in humans. However, in the light of the existence of a stringent system to control HTL formation in humans including the presence of homocysteine thiolactonase, bleomycin hydroxylase, paraoxonase, etc. in tissues, it appears that humans have employed certain protein sinks (HTL reservoir that is potential cross-linkers) as an additional strategy to control HTL toxicity^31,32^. To date, 2 proteins, serum albumin and transthyretin have been known to bind and carry HTL, but the binding does not exhibit a profound effect on protein functions^33,34^. The existence of such multiple protein sinks in the human will eventually open up new intellectual curiosities on the hyperhomocysteinemic fronts.

## Conclusion

We revealed that HTL-modified RNase-A and Lyz are completely resistant to the loss of functional and structural integrity. We also explored that the oligomer developed by them are functionally active. Functional oligomers are rare in nature. However, the existence of various functional amyloids e.g., curli fibers in quorum sensing and biofilm formation, fibrin polymerization in blood clotting, Mα amyloid in melanocytes, etc.^35^. Recently, the presence of large functional fractions in the inclusions formed by recombinantly-expressed proteins in bacteria has also been explored and their applications as natural immobilized biocatalysts or in tissue engineering have been provoked^35^. The native-like oligomer (explored in this study), therefore, further enlarged the repertoire of functional oligomers in humans. Nevertheless, the study indicates that it may not be necessary for all HTL-grown oligomers to become toxic or non-functional but may play a crucial role in sequestration of the HTL toxicity by acting as HTL protein-sink.

## Material

*Materials*- Commercially available lyophilized preparations of Lyz from chicken egg white (Cat. No. L6876), RNase-A from bovine pancreas (Cat. No. R5125), *Micrococcus lysodeikticus* (Cat no. M3770), and Cytidine 2′ 3′-cyclic monophosphate monosodium salt (Cat. No. C9630) were purchased from Sigma-Aldrich Company. HTL, ANS and ThT (Cat. No. T3516) were also purchased from Sigma. Ultra-pure samples of Guanidium Chloride (GdmCl) and other reagents were obtained from MP Biomedicals.

Protein samples of Lyz and RNase-A were dialyzed overnight against 0.1 M KCl (4 °C; pH-7.0) and protein stock solutions were filtered using 0.22 µM syringe filters. The stock solution of ANS was prepared using a molar extinction coefficient of 26,620 M^−1^ cm^−1^ at 416 nm. All other chemicals were of analytical grade and hence used without further purification. All solutions for optical measurements were prepared in the degassed 0.05 M Phosphate buffer (pH 7.0) containing 0.1 M KCl, using Double-Distilled water as an aqueous phase.

## Methods

### Determination of protein concentration and modification of proteins

The concentration of protein solutions was determined using the molar absorption coefficient, ε (M^−1^ cm^−1^), value of 3.9 × 10^4^ at 280 nm for Lyz, and 9800 at 277.5 nm for RNase-A. Lyz (2 mg/ml) and RNase-A (2 mg/ml) were treated with 0–1000 µM HTL in 0.05 M potassium phosphate buffer, pH 7.4 at 37 °C in a reaction volume of 1 ml. Protein samples were incubated for up to 7 days or as required. Aliquots of these samples were taken out for analysis at different time intervals. The modifications have been performed at least in triplicate.

### Estimation of the total –SH content using Ellman’s reagent

Protein sulfhydryl (SH) group estimation was carried out as described previously^15^. Briefly, fractions containing unmodified and modified proteins were solubilized in 6 M guanidinium hydrochloride in presence of 2 mM β-mercaptoethanol (ME) and incubated for 1 h at 37 °C as described earlier. Thereafter, to remove unbound HTL, precipitation was performed using 10% TCA. Precipitated protein was washed at least 3 times and resolubilized in phosphate buffer, pH 7.0. Total sulfhydryl content was calculated using 5, 5′-Dithiobis (2-nitrobenzoic acid), the Ellman's reagent as it absorbs at 412 nm. The amount of 5′-nitrothiobenzoate released was estimated from the molar extinction coefficient (*ε*) of 13,700 M^−1^ cm^−1^.

### Measurement and estimation of aggregation kinetic parameters

To measure the aggregation kinetic curves, both RNase-A and Lyz were treated with 1 mM HTL and incubated at different time intervals as indicated in the figures. We then added ThT dye to the modified samples and measured the ThT fluorescence at 485 nm. A plot between the relative fluorescence versus time was constructed. The aggregation kinetic parameters (*t*_lag_, *k*_app,_ and *I*_*f*_) were determined by analyzing the time-dependent curves using the following equation,

1 $$I = I_{0} + \frac{{I_{f} }}{{1 + e^{{ - \left( {\frac{{t - t_{0} }}{b}} \right)}} }}$$

where *I* is the fluorescence intensity at time *t*, and *t*_o_ is the time at 50% maximal light scattering. *I*_o_ represents the fluorescence intensity of the initial baseline and *I*_*f*_ represents the fluorescence intensity of the final plateau line, respectively. *b* is a constant. The apparent rate constant, *k*_app_ for the formation of aggregates is given by 1/*b,* and lag time (*t*_*agg)*_ is given by *t*_o_-2*b*^36,37^. Each curve was independently analyzed for the respective kinetic parameters and the mean was calculated.

### TEM imaging

Transmission electron micrographs were recorded on FEI Tecnai G2-200 kV HRTA transmission electron microscopy (Netherland) (equipped with digital imaging) facility available at All India Institute of Medical Sciences (AIIMS), New Delhi. Modified protein solutions (incubated for 7 days with 1000 µM HTL) were placed on a copper grid and air-dried. Negative staining was done by adding 1% uranyl acetate solution onto the copper grid before drying the sample and digital images were visualized and saved.

### Dynamic light scattering (DLS) measurements

Hydrodynamic radii of the modified proteins were measured using a Zetasizer Micro V/ZMV 2000 (Malvern, UK). The protein concentration used was 1.0 mg/ml. Measurements were made at a fixed angle of 90° using an incident laser beam of 689 nm. Fifteen measurements were made with an acquisition time of 30 s for each sample at a sensitivity of 10%. The data was analyzed using Zetasizer software provided by the manufacturer to get hydrodynamic diameters. The data was then analyzed to get hydrodynamic radii and polydispersity which is a measure of the standard deviation of the size of the particle.

### Enzyme activity measurements

Measurements of the functional activity of RNase-A were carried out using the procedure described by Crook et al.^38^*.* Briefly, the RNase-A-mediated hydrolysis of cytidine 2′–3′ cyclic monophosphate (C > p) was carried out for 60 min and the absorbance was monitored at 292 nm in a Jasco V-660 UV/Vis spectrophotometer. Substrate and protein concentrations used were 0.4 mg/ml and 0.035 mg/ml respectively.

For measurement of Lysozyme enzyme activity, *M. lysodeikticus* cell wall was used as a substrate as described by Mourel et al.^39^. Lysis of the bacterial cell wall in presence of Lyz was carried out for 1 h and the decrease in light scattering was recorded at 450 nm in a Jasco V-660 UV/Vis spectrophotometer with constant stirring. All experiments have been performed in triplicates and average errors were calculated manually. The results are expressed as percent activity keeping the enzyme activity unmodified samples at 100%.

### CD spectral measurements

Far-UV CD spectra of the modified protein samples were measured in a J-810 Jasco spectropolarimeter (equipped with a Peltier-type temperature controller). Each spectrum was corrected for the contribution of respective blanks. The concentration of the protein used was 0.5 mg/ml. Estimation of the individual components of the secondary structure was performed using the algorithm developed by Yang et al.^40^.

### Fluorescence spectral measurements

Fluorescence spectra of the modified protein samples were measured in Perkin Elmer LS 55 Spectrofluorimeter using a 3 mm quartz cell, with excitation and emission slits set at 10 nm. Protein concentration for all experiments was 5 µM for both proteins. For Tyr fluorescence measurements, in case of RNase-A excitation wavelength used was 268 nm, and emission was recorded from 290 to 450 nm. For Lyz, Trp was excited at 295 nm and emission was recorded in the wavelength region 310–500 nm. For ANS binding studies, ANS concentration was kept 16 times that of the protein working concentration. ANS was excited at 360 nm and emission was collected at 400–600 nm range. For ThT binding assay, samples were excited at 450 nm and emissions were collected in the wavelength range of 475–570 nm. The ThT concentration used was 25 µM.

### Measurement of light scattering intensity

For the measurement of light scattering intensity, 0.2 mg/ml of the protein samples were incubated with HTL in 0.05 M potassium phosphate buffer (pH 7.0). The change in light scattering intensity of the modified protein samples was monitored at 500 nm in a Jasco V-660 UV/Visible spectrophotometer equipped with a Peltier-type temperature controller. Measurements were repeated three times.

### MTT reduction assay

To evaluate the cytotoxic effect of the oligomers induced by RNase-A and Lyz on cell proliferation, MTT assay was performed. HeLa cells (5 × 104 cells/ml) were seeded onto a 96-well plate and allowed to grow for 24 h. After 24 h, cells were treated with different concentrations of the protein oligomers (10 µM and 15 µM for both the proteins) for another 24 h and 20 µl of MTT reagent (5 mg/ml) was added to each well. After incubation for 3 h at 37 °C, media was discarded from each well and 50 μl of DMSO was added to dissolve the formazan crystals. After incubation for 10 min, at 37 °C, absorbance was measured at 570 nm using an Elisa Plate-reader (Biotech, USA).

### DTT reduction assay

To check if the oligomers formed by Lyz (2 mg/ml) and RNase-A (2 mg/ml) upon HTL modification are disulfide cross-linked and reversible, the HTL-modified proteins were treated with reducing agent, dithiothreitol (0.8 mg/ml) and incubated for 3 h at 37 °C. We then analysed magnitude of the oligomers by measuring light scattering intensity. Three independent replicates were run.

### Statistical analysis

All the experiments were performed at least in triplicates and mean of the three independent measurements have been calculated and standard errors have been analysed. The standard errors are incorporated as error bars or expressed as ± standard derivative. *p*-values were calculated with one-way ANOVA using graph-pad prism software.

## Supplementary Information

Supplementary Information 1.

Supplementary Information 2.

Supplementary Information 3.

Supplementary Information 4.

Supplementary Information 5.

## Data Availability

All data generated or analysed during this study are included in this published article [and its supplementary information files].
