# Supplementary figures and images for: Functionally active cross-linked protein oligomers formed by homocysteine thiolactone
Source: Sci Rep. 2023 Apr 6;13:5620. doi: 10.1038/s41598-023-32694-2 (PMC10079695; doi:10.1038/s41598-023-32694-2)

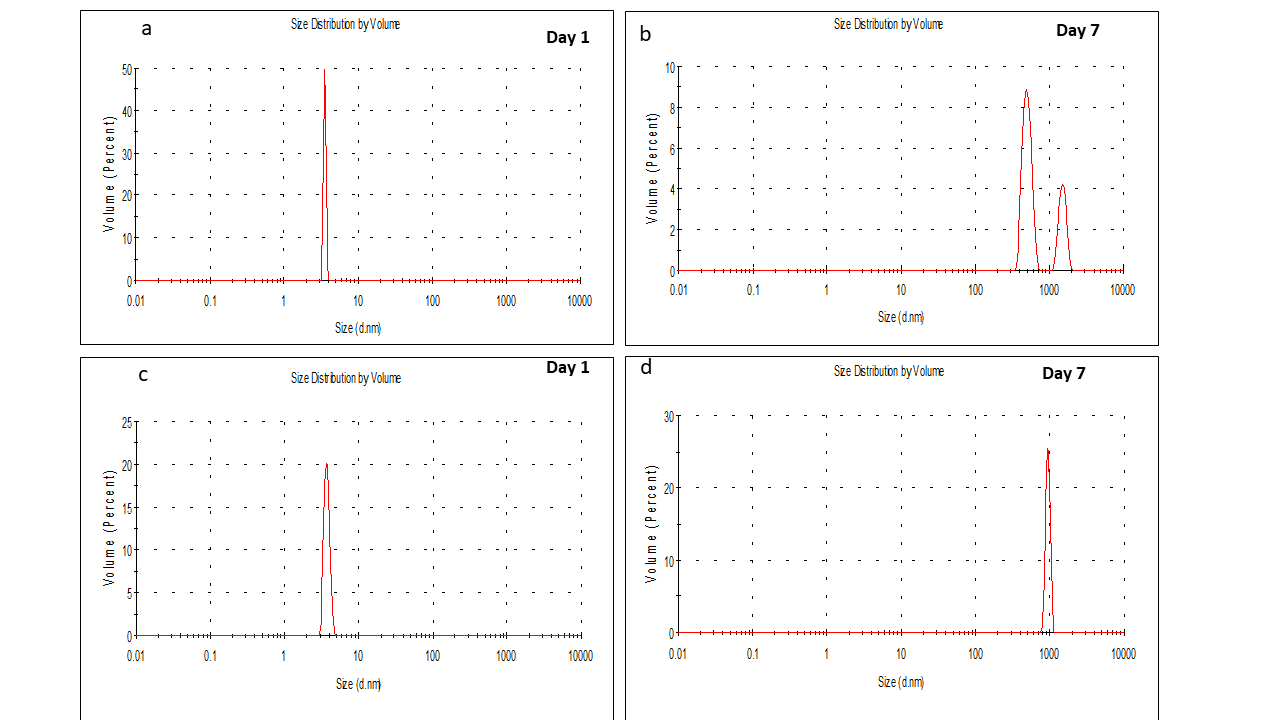

Supplement: Supplementary file 1 — Supplementary Information 1. [file 41598_2023_32694_MOESM1_ESM.tif]
