# Supplementary material for: Functionally active cross-linked protein oligomers formed by homocysteine thiolactone
Source: Sci Rep. 2023 Apr 6;13:5620. doi: 10.1038/s41598-023-32694-2 (PMC10079695; doi:10.1038/s41598-023-32694-2)
Supplement: Supplementary file 3 — Supplementary Information 3. [file 41598_2023_32694_MOESM3_ESM.docx]

**SUPPLEMENTARY FIGURES**

**Figure S1:** **Representative DLS profiles of HTL-modified RNase-A and Lyz:** Size by volume distribution of HTL-modified RNase-A (upper panels) and Lyz (lower panels) on day 1 and day 7.

**Figure S2: Different secondary structural elements of HTL-modified RNase-A and Lyz:** Percent α, β, and random coil components of RNase-A **(left panel)** and Lyz **(right panel)** upon modification with 1000µM HTL.

**SUPPLEMENTARY TABLES**

**Table S1. Aggregation kinetic parameters of HTL-modified proteins:** Measured kinetic parameters, *t*_lag_, *k*_app_ and *I_f_* of the modified proteins estimated using equation 1.

**Table S2. Total sufhydryl content of HTL modified proteins:** Day dependent -SH content (mol/mol x 1000) of RNase-A and Lyz upon covalent modification with 1000 µM HTL.
