# Supplementary material for: Functionally active cross-linked protein oligomers formed by homocysteine thiolactone
Source: Sci Rep. 2023 Apr 6;13:5620. doi: 10.1038/s41598-023-32694-2 (PMC10079695; doi:10.1038/s41598-023-32694-2)
Supplement: Supplementary file 4 — Supplementary Information 4. [file 41598_2023_32694_MOESM4_ESM.docx]

**Table S1**

|  | ***t*_lag_**  **[Hours]** | ***K*_app_**  **[Hours^-1^]** | ***I*_f_** |
| --- | --- | --- | --- |
| **RNase-A** | 76.5 | 0.073 | 133 |
| **Lyz** | 83 | 0.072 | 173.81 |
