# Supplementary material for: Functionally active cross-linked protein oligomers formed by homocysteine thiolactone
Source: Sci Rep. 2023 Apr 6;13:5620. doi: 10.1038/s41598-023-32694-2 (PMC10079695; doi:10.1038/s41598-023-32694-2)
Supplement: Supplementary file 5 — Supplementary Information 5. [file 41598_2023_32694_MOESM5_ESM.docx]

|  | **RNase-A** | | **Lyz** | |
| --- | --- | --- | --- | --- |
| **No. of days** | **-HTL** | **+HTL** | **-HTL** | **+HTL** |
| 0 | 74.59 ±8.10 | 243.01±10.80 | 102.89±14.40 | 201.61±18.00 |
| 1 | 88.09±12.82 | 911.30 ± 45.56 | 142.64±2.16 | 612.039± 21.60 |
| 2 | 101.72±13.50 | 1383.82 ± 19.57 | 145.88±18.72 | 1216.87± 30.24 |
| 3 | 224.78±12.15 | 1745.64±59.20 | 168.13±56.16 | 2066.53±26.64 |
| 4 | 317.94±24.30 | 2442.95±54.00 | 346.84±11.52 | 2325.74±67.68 |
| 5 | 415.14±33.75 | 3195.62±62.10 | 507.99±41.76 | 2930.58±66.96 |
| 6 | 350.34±14.17 | 3867.28±20.25 | 424.25±.31.68 | 3220.04±45.36 |
| 7 | 310.51±23.62 | 3820.71±41.85 | 455.50±14.40 | 3248.05±30.24 |
